# Human Adenovirus Surveillance — United States, 2003–2016

**DOI:** 10.15585/mmwr.mm6639a2

**Published:** 2017-10-06

**Authors:** Alison M. Binder, Holly M. Biggs, Amber K. Haynes, Christina Chommanard, Xiaoyan Lu, Dean D. Erdman, John T. Watson, Susan I. Gerber

**Affiliations:** 1 Division of Viral Diseases, National Center for Immunization and Respiratory Disease, CDC.

Human adenoviruses (HAdVs) are nonenveloped, double-stranded DNA viruses in the family Adenoviridae; seven species (A–G) and >60 genotypes are known to cause human infection (*1*). Clinical manifestations associated with HAdV infection include fever, acute respiratory illness, gastroenteritis, and conjunctivitis. HAdV infection can be severe, particularly among immunocompromised patients, and can cause respiratory failure, disseminated infection, hemorrhagic cystitis, neurologic disease, and death (*1*,*2*). Illness tends to occur sporadically and without demonstrated seasonality. Outbreaks of HAdV have been reported globally in communities (*3*), and in closed or crowded settings, including dormitories, health care settings, and among military recruits, for whom a vaccine against HAdV type 4 (HAdV-4) and HAdV type 7 (HAdV-7) has been developed (*4*,*5*). CDC summarized HAdV detections voluntarily reported through the National Adenovirus Type Reporting System (NATRS) after initiation of surveillance in 2014 to describe trends in reported HAdVs circulating in the United States. Reporting laboratories were also encouraged to report available results for specimens collected before surveillance began. Overall, the number of reporting laboratories and HAdV type identifications reported to NATRS has increased substantially from the start of official reporting in 2014 through 2016; this report describes specimens collected during 2003–2016. The most commonly reported HAdV types were HAdV type 3 (HAdV-3) and HAdV type 2 (HAdV-2), although HAdV types reported fluctuated considerably from year to year. In the United States, information on recently circulating HAdV types is needed to inform diagnostic and surveillance activities by clinicians and public health practitioners. Routine reporting to NATRS by all U.S. laboratories with the capacity to type HAdVs could help strengthen this surveillance system.

NATRS is a passive laboratory-based surveillance system initiated in 2014 to coordinate reporting of laboratory identifications of HAdV types in the United States. Traditional typing techniques based on serologic methods have been largely replaced by molecular typing techniques including sequencing and conventional or real-time polymerase chain reaction, which can rapidly determine HAdV types. However, the number of public health and clinical laboratories with the capacity to type HAdVs using molecular techniques remains limited, and traditional methods are labor intensive and time consuming. Commercial molecular assays are more readily available; although some of these assays determine species, they do not identify specific types. Public health and clinical laboratories without the capacity to type HAdVs can send specimens of public health or clinical importance to CDC’s Respiratory Virus Diagnostics Laboratory. All participating laboratories are encouraged to report HAdV typing data quarterly to NATRS accompanied by limited demographic, clinical, and laboratory data.

Eleven laboratories reported data to NATRS during 2014–2016, including the CDC Respiratory Virus Diagnostics Laboratory, public health laboratories from seven states, two clinical laboratories, and one U.S. Department of Defense laboratory. Data with specimen-collection years 2003–2013 represent retrospective data from reporting laboratories with the capacity to test and type before surveillance formally began in 2014. All typing data for specimens collected during 2003–2007 was provided by the CDC Respiratory Virus Diagnostics Laboratory. Five laboratories, including the CDC Respiratory Virus Diagnostics Laboratory, provided data for typed detections among specimens collected during 2008–2013 (ranging from two laboratories in 2008 to five in 2013). NATRS received reports for 2,138 HAdV detections among specimens collected during 2003–2016. Species and type were reported for 2,107 (98.6%) and 1,497 (70.0%) detections, respectively, representing 22 types (Table 1) from 32 states and the U.S. Virgin Islands, according to patient state of residence. The most commonly reported specimen types were respiratory specimens (N = 1,227; 82.0%), ocular swabs (61; 4.1%), and stool or rectal swabs (35; 1.8%). Species C (N = 683; 41%) and B (657; 40%) were the most commonly reported species during the 13-year period. The number of typed HAdV identifications reported by year of specimen collection ranged from two in 2003 to 269 in 2014 when data collection began and increased to 362 in 2016. HAdV-3 (N = 341; 22.8%), HAdV-2 (293; 19.6%), HAdV type 1 (HAdV-1) (248; 16.6%), HAdV-4 (185; 12.4%), HAdV-7 (127; 8.5%), and HAdV type 14 (HAdV-14) (89; 5.9%) accounted for 1,283 (85.5%) of the 1,497 reports with type identified (Figure) (Table 1). A single type was identified in 1,490 (99.5%) specimens, whereas detection of two types was identified in seven (0.5%) specimens. Year-to-year fluctuations in circulating types were evaluated using data reported during 2014–2016. HAdV-3, HAdV-2, HAdV-1, and HAdV-4 were among the most common types identified each year during this period; however, the frequency with which individual types were reported varied considerably from year to year (Table 2). The most commonly reported typing methods included serum neutralization (N = 591; 39.5%), full or partial genome sequencing (567; 37.9%), and real-time polymerase chain reaction (285; 19.0%).

**TABLE 1:** Number and percentage of human adenovirus (HAdV) detections, by species and type — National Adenovirus Type Reporting System, 32 states and the U.S. Virgin Islands, 2003–2016

HAdV species	HAdV type	No. (%) of detections
**A**	**12**	** t3 (0.2)**
	31	3 (0.2)
B	3*	341 (22.8)
	7*	127 (8.5)
	11	6 (0.4)
	14*	89 (5.9)
	21	34 (2.3)
	34	2 (0.1)
	35	14 (0.9)
C	1*	248 (16.6)
	2*	293(19.6)
	5	56 (3.7)
	6	20(1.3)
D	8	54 (3.6)
	15	1 (0.1)
	19	1 (0.1)
	22	1 (0.1)
	29	1 (0.1)
	37	12 (0.8)
	56	1 (0.1)
E	4*	185 (12.4)
F	41	5 (0.3)
**Total**	**22**	**1,497 (100)**

* One of the six most common types detected, accounting for 1,283 (85.5%) of reports.

**FIGURE:**
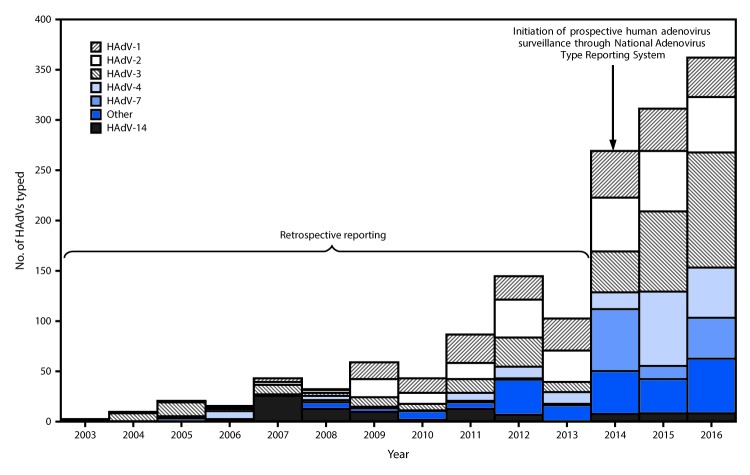
Distribution of human adenovirus species (HAdVs) and types, by year of specimen collection* — National Adenovirus Type Reporting System, 32 U.S. states and the U.S. Virgin Islands, 2003–2016 * Frequency of the most common HAdV types reported after surveillance initiation in 2014 varied by year of specimen collection.

**TABLE 2:** Number and percentage of human adenovirus detections, by species, type, and year of specimen collection* — National Adenovirus Type Reporting System, 32 states and the U.S. Virgin Islands, 2003–2016

Retrospectively reported to NATRS		Reported to NATRS after surveillance initiation						Total NATRS 2003–2016 (N = 1,497)	
2003–2013 (n = 555)		2014 (n = 269)		2015 (n = 311)		2016 (n = 362)			
Species/Type	No. (%)	Species/Type	No. (%)	Species/Type	No. (%)	Species/Type	No. (%)	Species/Type	No. (%)
**C1**	**124 (22.3)**	**B7**	**62 (23)**	**B3**	**80 (25.7)**	**B3**	**115 (31.8)**	B3	341 (22.8)
**B3**	**121 (21.8)**	**C2**	**54 (20.1)**	**E4**	**74 (23.8)**	**C2**	**55 (15.2)**	C2	293 (19.6)
**C2**	**105 (18.9)**	**C1**	**46 (17.1)**	**C2**	**60 (19.3)**	**E4**	**50(13.8)**	C1	248 (16.6)
**D8**	**66 (11.9)**	**B3**	**41 (15.2)**	**C1**	**42 (13.5)**	**B7**	**41 (11.3)**	E4	185 (12.4)
**B14**	**45 (8.1)**	**E4**	**16 (5.9)**	**C5**	**17 (5.5)**	**C1**	**39 (10.8)**	B7	127 (8.5)
B7	33 (5.9)	C5	14 (5.2)	B7	13 (4.2)	B21	15 (4.1)	**B14**	**89 (5.9)**
C5	16 (2.9)	B21	11 (4.1)	B14	8 (2.6)	D8	13 (3.6)	**C5**	**56 (3.7)**
E4	11 (2)	B14	7 (2.6)	C6	7 (2.3)	C5	9 (2.5)	**D8**	**54 (3.6)**
B21	11 (2)	D8	5 (1.9)	D8	3 (1)	B14	8 (2.2)	**B21**	**34 (2.3)**
A12	6 (1.1)	D37	5 (1.9)	B21	3 (1)	C6	6 (1.7)	**C6**	**20 (1.3)**
B35	5 (0.9)	C6	4 (1.5)	D37	2 (0.6)	B35	3 (0.8)	**B35**	**14 (0.9)**
B11	3 (0.5)	A31	2 (0.7)	F41	1 (0.3)	D37	3 (0.8)	**D37**	**12 (0.8)**
F41	3 (0.5)	D19	1 (0.4)	D15	1 (0.3)	F41	2 (0.6)	**B11**	**6 (0.4)**
C6	2 (0.4)	D22	1 (0.4)	B35	—^†^	D29	1 (0.3)	**F41**	**5 (0.3)**
D37	2 (0.4)	B35	—^†^	A12	—^†^	B34	1 (0.3)	**A12**	**3 (0.2)**
A31	1 (0.2)	F41	—^†^	A31	—^†^	D56	1 (0.3)	**A31**	**3 (0.2)**
B34	1 (0.2)	A12	—^†^	B11	—^†^	A12	—^†^	**B34**	**2 (0.1)**
D15	—^†^	B11	—^†^	D19	—^†^	A31	—^†^	**D15**	**1 (0.1)**
D19	—^†^	D15	—^†^	D22	—^†^	B11	—^†^	**D19**	**1 (0.1)**
D22	—^†^	D29	—^†^	D29	—^†^	D15	—^†^	**D22**	**1 (0.1)**
D29	—^†^	B34	—^†^	B34	—^†^	D19	—^†^	**D29**	**1 (0.1)**
D56	—^†^	D56	—^†^	D56	—^†^	D22	—^†^	**D56**	**1 (0.1)**

**Abbreviation**: NATRS = National Adenovirus Type Reporting System.

* HAdV types reported after frequency of the most common surveillance initiation in 2014 varied by year of specimen collection.

^†^ Data not reported.

## Discussion

Type-based HAdV surveillance in the United States has three objectives: 1) to monitor patterns of circulation for HAdV types over time; 2) to assist with recognition and confirmation of outbreaks associated with circulating types; and 3) to inform development or use of diagnostics tests, therapeutics, and vaccines. After initiation of NATRS in 2014, this is the first report describing national trends in HAdV type circulation in the United States, and fluctuations in frequency of HAdV types during 2014–2016.

During 2003–2016, the six most commonly reported types were HAdV-3, HAdV-2, HAdV-1, HAdV-4, HAdV-7, and HAdV-14, which have all been detected in association with acute respiratory illness worldwide (*2*). HAdV-7 was recognized in the United States as an important cause of severe respiratory illness among adults in a community outbreak in Oregon in 2014 (*3*). Nearly half of HAdV-7 detections (n = 62; 48.8%) in NATRS occurred during 2014 (Table 2) as a result of sampling and typing of specimens collected as part of the Oregon outbreak investigation. Before 2014, HAdV-7 was uncommonly reported among civilian populations, although respiratory illness outbreaks had been reported among military recruits (*6*). Respiratory illness associated with HAdV-4 also has been well documented among military recruits, but has been less commonly reported among civilian populations (*7*).

A live, oral vaccine against HAdV-4 and HAdV-7 was given to all U.S. military members from 1971 to 1999. After depletion of the vaccine in 1999, HAdV-4 reemerged as the main cause of febrile respiratory illness among military service members, especially among those in initial entry (i.e., basic) training. Subsequent reintroduction of HAdV-4 and HAdV-7 vaccine at all U.S. initial entry training sites in late 2011 led to declines in overall rates of respiratory illness and in incidence of adenovirus infections(*8*,*9*). The prevalence of respiratory illness associated with HAdV-4 and HAdV-7 in non-military congregate populations that might potentially benefit from HAdV vaccination is not known.

Other common HAdV types reported to NATRS include HAdV-14, which was first documented in North American military populations in 2006 (*2*,*10*). After HAdV-4 and HAdV-7 vaccine re-introduction in 2011, HAdV-14 surpassed HAdV-4 and HAdV-7 as the most prevalent HAdV type reported in these settings, although the actual number of cases of HAdV-14 did not increase (*8*). By 2007, outbreaks of respiratory illness because of HAdV-14 in multiple civilian populations were also documented (*4*). Among the 89 HAdV-14 detections reported to NATRS during 2003–2016, the highest number (25) and percentage (28.1%) were reported during 2007, likely coinciding with specimens collected during documented outbreaks of HAdV-14 among community and military populations and in health care settings in certain states during this period.

The findings in this report are subject to at least four limitations. First, NATRS is a passive system that relies on voluntary participation from laboratories, and data might be biased by outbreak investigations and nonrandom sample selection for typing; therefore, types reported might not be representative of the HAdV types circulating nationally or regionally. Second, NATRS collects limited clinical information, restricting the interpretation of trends in HAdV disease associated with circulating types. Third, although quarterly reporting to NATRS is encouraged and allows for retrospective outbreak documentation, not all participating laboratories submit timely data, limiting the ability to detect outbreaks of HAdVs in real-time. Finally, although the number of laboratories with the capacity to test for specific HAdV types and report to NATRS is increasing, the relatively small number of reporters limits the reliability and generalizability of these results.

NATRS monitors patterns of HAdV circulation in the United States based on voluntary laboratory reporting of detections by type. Understanding currently circulating HAdV types and improving the reliability and generalizability of surveillance data relies upon voluntary reports to NATRS from public health and clinical laboratories. The long-term sustainability of NATRS depends on building and maintaining the capacity to identify and type HAdVs among public health and clinical laboratories; improving timeliness of reporting by currently participating laboratories; and increasing the number of participating laboratories.

**Summary**

**What is already known about this topic?**

Human adenoviruses (HAdVs) can cause a wide spectrum of clinical illness, ranging from asymptomatic infections to severe illnesses and death. Approximately 60 HAdV genotypes have been identified to date, and they are associated with different clinical illnesses, including respiratory illness, gastroenteritis, and conjunctivitis. Surveillance for circulating HAdV types in the United States is passive and voluntary but might be useful to inform diagnostic and surveillance activities by clinicians and public health practitioners.

**What is added by this report?**

Based on data from the National Adenovirus Type Reporting System, the most commonly reported types of HAdVs during 2003–2016 in the United States were HAdV types 1, 2, 3, 4, 7, and 14, which accounted for 85.5% (n = 1,283) of all types reported. Year-to-year fluctuations in HAdV types circulating in the United States varied considerably, likely reflecting increases in testing in response to recognized HAdV outbreaks.

**What are the implications for public health practice?**

HAdV type-based surveillance data can be used to determine patterns of circulation for individual HAdV types in the United States, assist with the recognition and documentation of outbreaks associated with circulating types, and guide development of new diagnostic tests, therapeutics, and vaccines.
